# Supplementary material for: Brainstem BDNF neurons are downstream of GFRAL/GLP1R signalling
Source: Nat Commun. 2024 Dec 30;15:10749. doi: 10.1038/s41467-024-54367-y (PMC11685588; doi:10.1038/s41467-024-54367-y)
Supplement: Supplementary file 1 — Supplementary Information [file 41467_2024_54367_MOESM1_ESM.pdf]

Supplemental Information

Brainstem BDNF neurons are downstream of GFRAL/GLP1R signalling

Authors:

Claire H. Feetham[1]*, Valeria Collabolletta[1]*, Amy A. Worth[1], Rosemary Shoop[1], Sam Groom[1], Court Harding[1], Mehdi Boutagouga Boudjadja[1], Tamer Coskun[2], Paul J. Emmerson[2], Giuseppe D'Agostino[1] & Simon M. Luckman[1]

Affiliations:

[1]Faculty of Biology, Medicine and Health, University of Manchester, Manchester, UK.

[2]Lilly Research Laboratories, Eli Lilly & Company, Indianapolis, United States.

Contents of supplementary information:

Supplementary Figure 1

Supplementary Figure 2

Supplementary Figure 3

Supplementary Figure 4

Supplementary Figure 5

Supplementary Figure 6

Supplementary Figure 7

Supplementary Figure 1

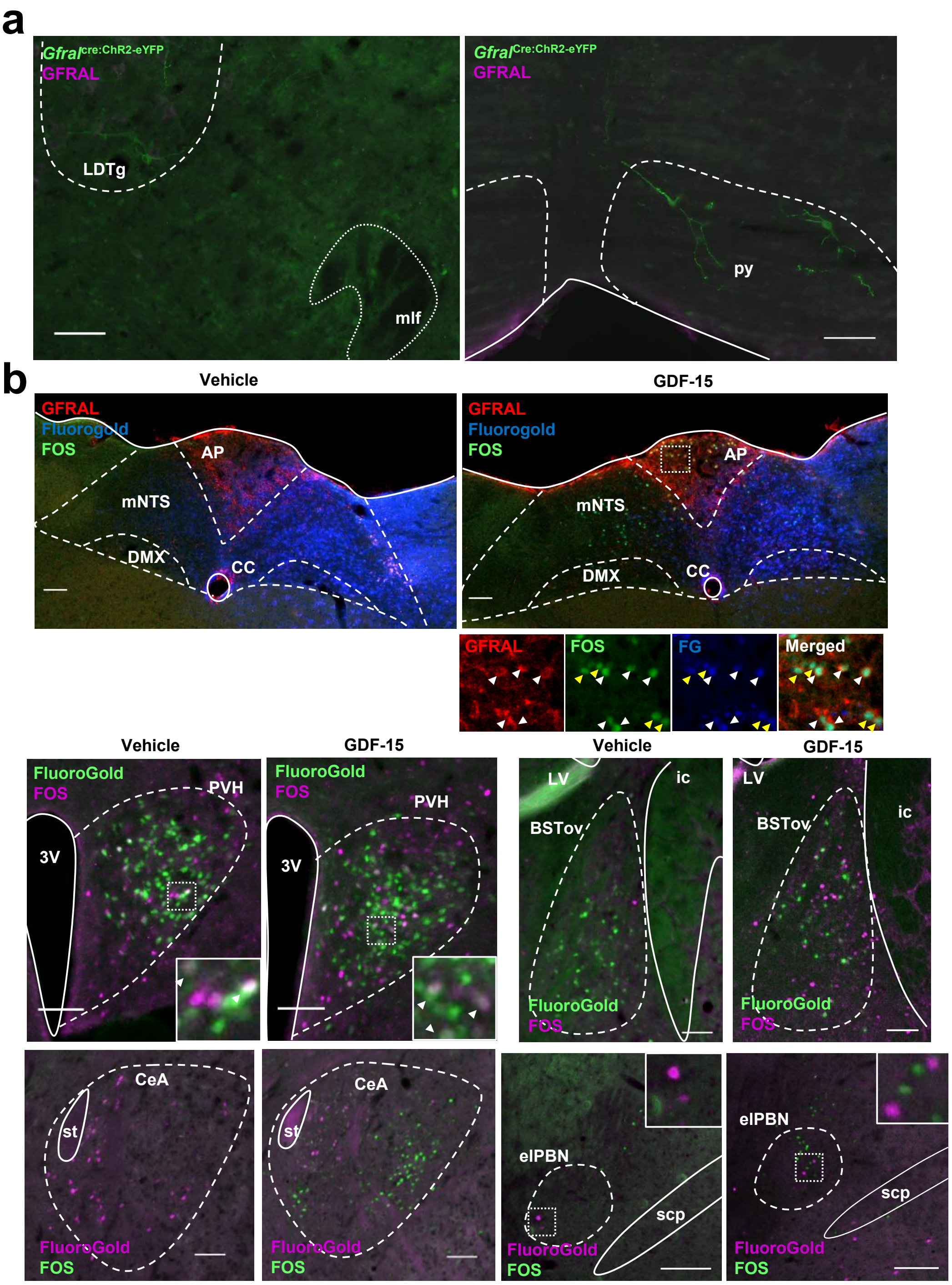

Supplementary Figure 1. Forebrain neurons activated by GDF15 do not send descending projections to the NTS. (**a**) Dual-fluorescence labelling for GFRAL (magenta) with eYFP (staining using antibody raised against green fluorescent protein) in *Gfral*$^{Cre:ChR2\text{-}eYFP}$ mice showing occasional eYFP+ve cell bodies in the laterodorsal tegmental nucleus and in the pyramidal tracts/olivary complex. (**b**) FluoroGold (FG) injections were made into the medial NTS (mNTS) of mice which were later injected with vehicle or GFD15 (n = 4 per group). Triple labelling for FG, native GFRAL and FOS demonstrated activated GFRAL neurons in the AP project to the medial NTS. White arrows in higher magnification insets indicate co-labelled cells. A small number of FOS-containing neurons in the PVH project to the mNTS, though

there was not an obvious increase after GDF15. White arrows indicate co-localisation of FluoroGold and FOS. By comparison, no GDF15-activated neurons in the elPBN, CeA or BSTov project directly to the mNTS. 3V (third ventricle), AP (area postrema), BSTov (bed nucleus of the stria terminalis, oval nucleus), CC (central canal), CeA (central amygdala), DMX (dorsal motor nucleus of the tenth cranial, vagus nerve), elPBN (exterolateral parabrachial nucleus), ic (internal capsule), LDTg (laterodorsal tegmental nucleus), LV (lateral ventricle), mlf (medial longitudinal fasciculus), PVH (paraventricular nucleus of the hypothalamus), py (pyramidal tract), scp (superior cerebellar peduncle, st (stria terminalis), Scale bars indicate 100 µm. Source data are provided as a Source Data file.

Supplementary Figure 2

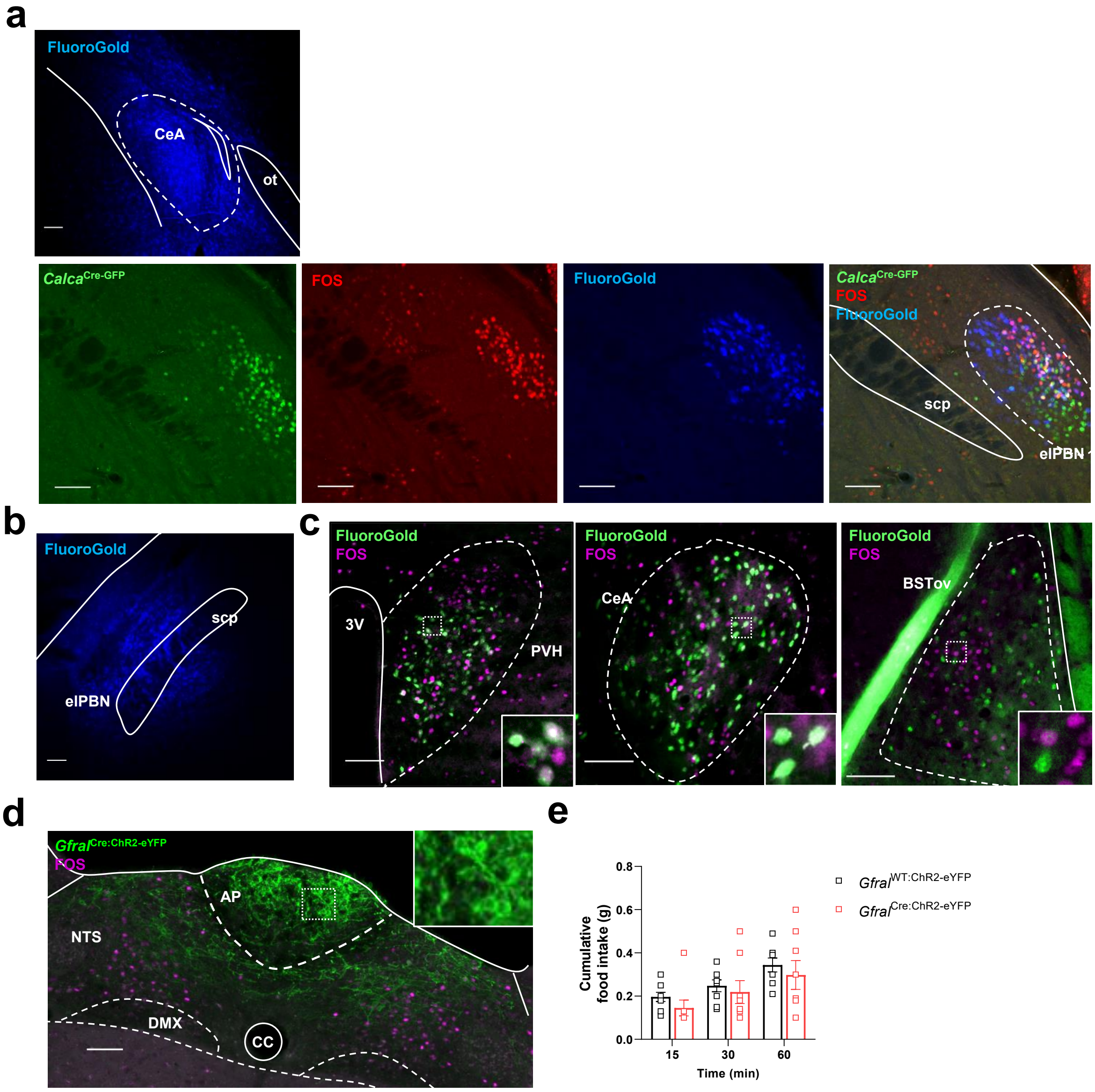

Supplementary Figure 2. GDF15 activates a GFRAL→$CGRP^{elPBN}$→CeA pathway. (**a**) Triple fluorescent staining showing, GDF15-activated CGRP neurons in the elPBN (bottom panel) projecting to the ipsilateral CeA, as demonstrated by FluoroGold (FG) retrotracer injected into the CeA of *Calca*$^{Cre\text{-}GFP}$ mice (top panel; n = 5). (**b**) FG was also injected into the elPBN of additional mice (n = 5). (**c**) Many cells from the PVH, CeA and BSTov project to the elPBN, though only a few of these are activated by GDF15. (**d**) Dual fluorescence labelling of *Gfral*$^{Cre:ChR2\text{-}eYFP}$ mice following optogenetic stimulation of GFRAL terminals in the elPBN. There is no FOS in the GFRAL cell bodies. (**e**) Cumulative food intake in overnight fasted *Gfral*$^{Cre:ChR2\text{-}eYFP}$ (red squares; n = 6) and *Gfral*$^{WT:ChR2\text{-}eYFP}$ (black squares; n = 8) mice after fibre tethering with no optogenetic stimulation (two-way ANOVA followed by Sidak's multiple comparisons test. Data presented as mean ± SEM). 3V (third ventricle), AP (area postrema), BSTov (bed nucleus of the stria terminalis, oval nucleus), CC (central canal), CeA (central amygdala), DMX (dorsal motor nucleus of the tenth cranial, vagus nerve), elPBN (exterolateral parabrachial nucleus), NTS (nucleus of the tractus solitarius), ot (optic tract), PVH

(paraventricular nucleus of the hypothalamus), scp (superior cerebellar peduncle). Source data are provided as a Source Data file.

Supplementary Figure 3

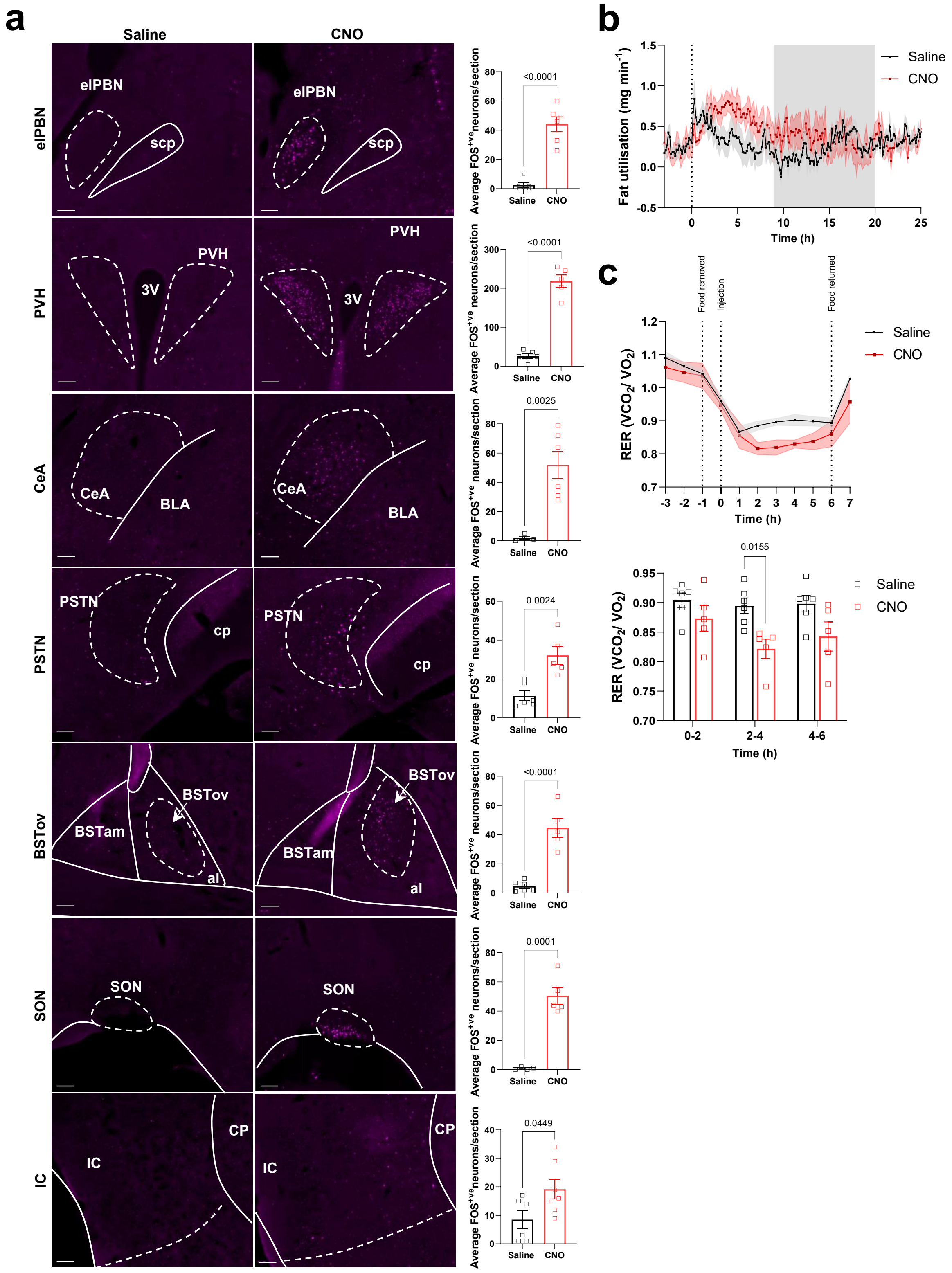

Supplementary Figure 3. Faithful activation of GFRAL neurons induces FOS in multiple brain regions and increases fatty acid oxidation. (**a**) Fluorescent staining of saline and CNO (3 mg kg^{-1}) induced FOS (magenta) in the elPBN, PVH, CeA, PSTN, BSTov, SON and IC of *Gfral*$^{Cre:hM3Dq\text{-}mCherry}$. The average FOS+ve neurons/section is presented on the right (saline

black squares, CNO red squares; n = 4-7 per group). elPBN ****p<0.0001, PVH ****p<0.0001, CeA **p=0.0025, PSTN **p=0.0024, BSTov ****p<0.0001, SON ****p<0.0001, IC *p=0.0449; comparisons made by two-tailed Student's unpaired t-tests. (**b**) Indirect calorimetry assessment of fat utilisation (mg min^{-1}) in *Gfral*$^{Cre:hM3Dq\text{-}mCherry}$ mice after saline (black squares) or CNO (red squares; n = 6 per group). (**c**) Indirect calorimetry assessment of RER in *Gfral*$^{Cre:hM3Dq\text{-}mCherry}$ mice after saline (black squares; n = 6) or CNO (red squares; n = 5) in the absence of food (*p=0.0155; two-way ANOVA with Sidak's multiple comparisons test). Data are presented as mean ± SEM. 3V (third ventricle), IC (infralimbic cortex), BSTal, am, ov (bed nucleus of the stria terminalis, anterolateral, medial lateral and oval nuclei), CeA (central amygdala), cp (cerebral peduncle), elPBN (external lateral parabrachial nucleus), PSTN (parasubthalamic nucleus), PVH (paraventricular nucleus of the hypothalamus), scp (superior cerebellar peduncle), SON (supraoptic nucleus of the hypothalamus). Scale bars indicate 100 µm. Source data are provided as a Source Data file.

Supplementary Figure 4

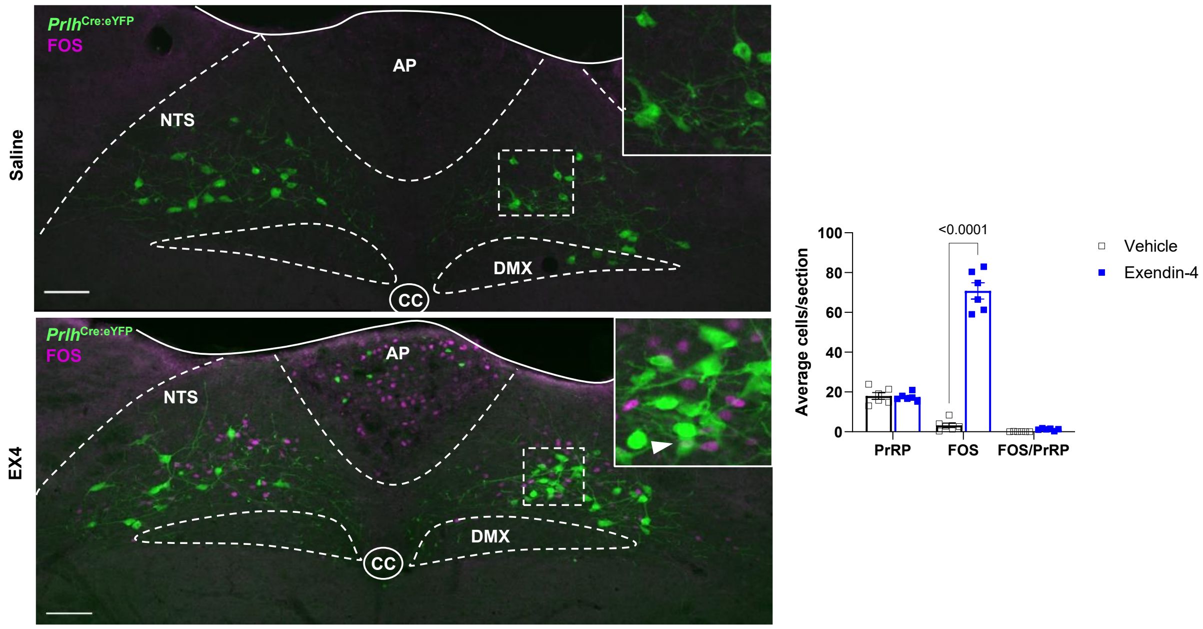

Supplementary Figure 4. EX4 does not induce FOS in PrRP (gene name *Prlh*) neurons. Dual fluorescent staining of FOS (magenta) with eYFP (staining using antibody raised against green fluorescent protein) in *Prlh*$^{Cre:eYFP}$ mice administered vehicle or EX4 (30 µg kg^{-1}). FOS is increased in the AP and NTS in EX4-treated mice, but not in *Prlh*$^{Cre:eYFP}$ neurons (green) (vehicle black squares, EX4 blue squares; n = 6 per group, ****$p<0.0001$, two-way ANOVA with Sidak's multiple comparisons test). Data are presented as mean ± SEM. AP (area postrema), CC (central canal), DMX (dorsal motor nucleus of the tenth cranial, vagus nerve), NTS (nucleus of the tractus solitarius). Scale bars indicate 100 µm. Source data are provided as a Source Data file.

Supplementary Figure 5

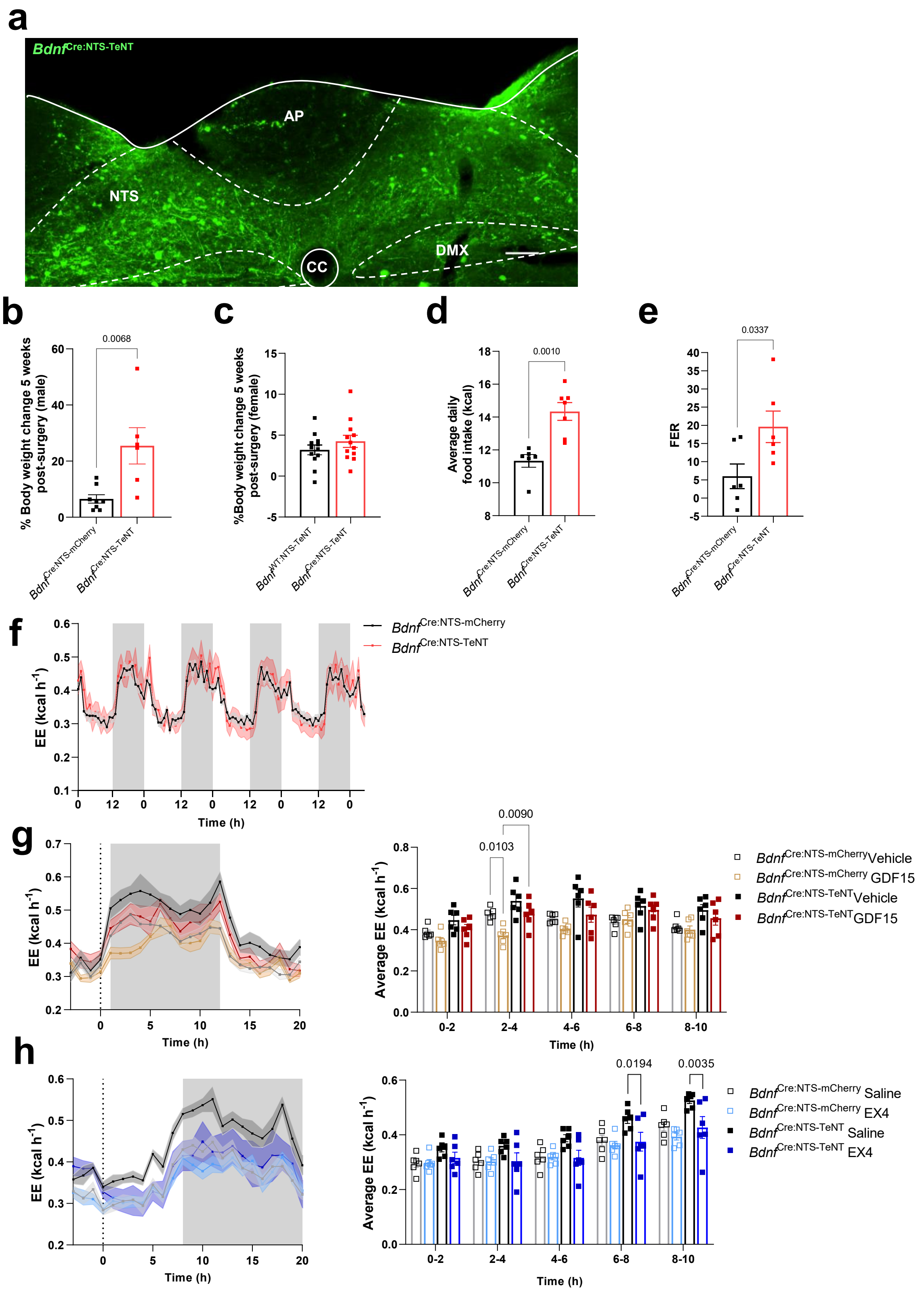

Supplementary Figure 5. Metabolic effects of disabling BDNF^{NTS} neurons. (**a**) Representative immunofluorescence image of AAV-TeNT (in green) expression in BDNF^{NTS} neurons of *Bdnf*^{cre} mice. Scale bar indicates 100 μm. Percentage body weight change in

Bdnf$^{Cre:NTS-TeNT}$ (red squares; n = 6) and *Bdnf*$^{Cre:NTS-mCherry}$ (black squares; n = 8) male (**b**) and female (n = 12 per group) (**c**) mice 5 weeks after viral injection (**p=0.0068, comparisons made by two-tailed Student's unpaired t-test). (**d**) Average daily food intake and (**e**) food efficiency ratio (FER) of *Bdnf*$^{Cre:NTS-TeNT}$ (red squares) and *Bdnf*$^{Cre:NTS-mCherry}$ (black squares) mice calculated as body weight gain (g) per kcal consumed (n = 6 per group; ***p=0.0004 and *p=0.0337 respectively, comparisons made by two-tailed Student's unpaired t-test). (**f**) Indirect calorimetry assessment of energy expenditure in *Bdnf*$^{Cre:NTS-TeNT}$ (red squares) and *Bdnf*$^{Cre:NTS-mCherry}$ (black squares) mice (n = 6 per group). Energy expenditure in same mice following treatment with (**g**) GDF15 (*Bdnf*$^{Cre:NTS-mCherry}$ vehicle black open squares, GDF15 brown open squares; *Bdnf*$^{Cre:NTS-TeNT}$ vehicle black closed squares, GDF15 red closed squares; *p=0.0103, **p=0.0090; two-way ANOVA with Tukey's multiple comparisons test) or (**h**) EX4 (*Bdnf*$^{Cre:NTS-mCherry}$ saline black open squares, EX4 light blue open squares; *Bdnf*$^{Cre:NTS-TeNT}$ saline black closed squares, EX4 dark blue closed squares; *p=0.0194 **p=0.0035; two-way ANOVA with Tukey's multiple comparisons test). AP (area postrema), CC (central canal), DMX (dorsal motor nucleus of the tenth cranial, vagus nerve), NTS (nucleus of the tractus solitarius). Source data are provided as a Source Data file.

Supplementary Figure 6

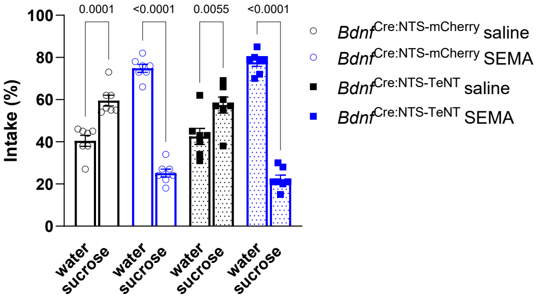

Supplementary Figure 6. BDNF NTS neurons are not required for GLP1RA-induced aversion. Intraperitoneal injection of the GLP1RA, semaglutatide (SEMA; 10 nmol kg^{-1}), causes aversion in both *Bdnf*$^{\text{Cre:NTS-TeNT}}$ and *Bdnf*$^{\text{Cre:NTS-mCherry}}$ mice, as measured by a conditioned taste avoidance (CTA) test. Semaglutide treatment is paired with sucrose as a conditioned stimulus in a two-bottle CTA. Water and sucrose intake are measured following conditioning (*Bdnf*$^{\text{Cre:NTS-TeNT}}$ saline and SEMA black closed squares and blue closed squares respectively, *Bdnf*$^{\text{Cre:NTS-mCherry}}$ saline and SEMA black open circles and blue open circles respectively; n = 7 per group; **p=0.0055, ***p=0.0001, ****p<0.0001; two-way ANOVA with Tukey's multiple comparisons test). Source data are provided as a Source Data file.

Supplementary Figure 7

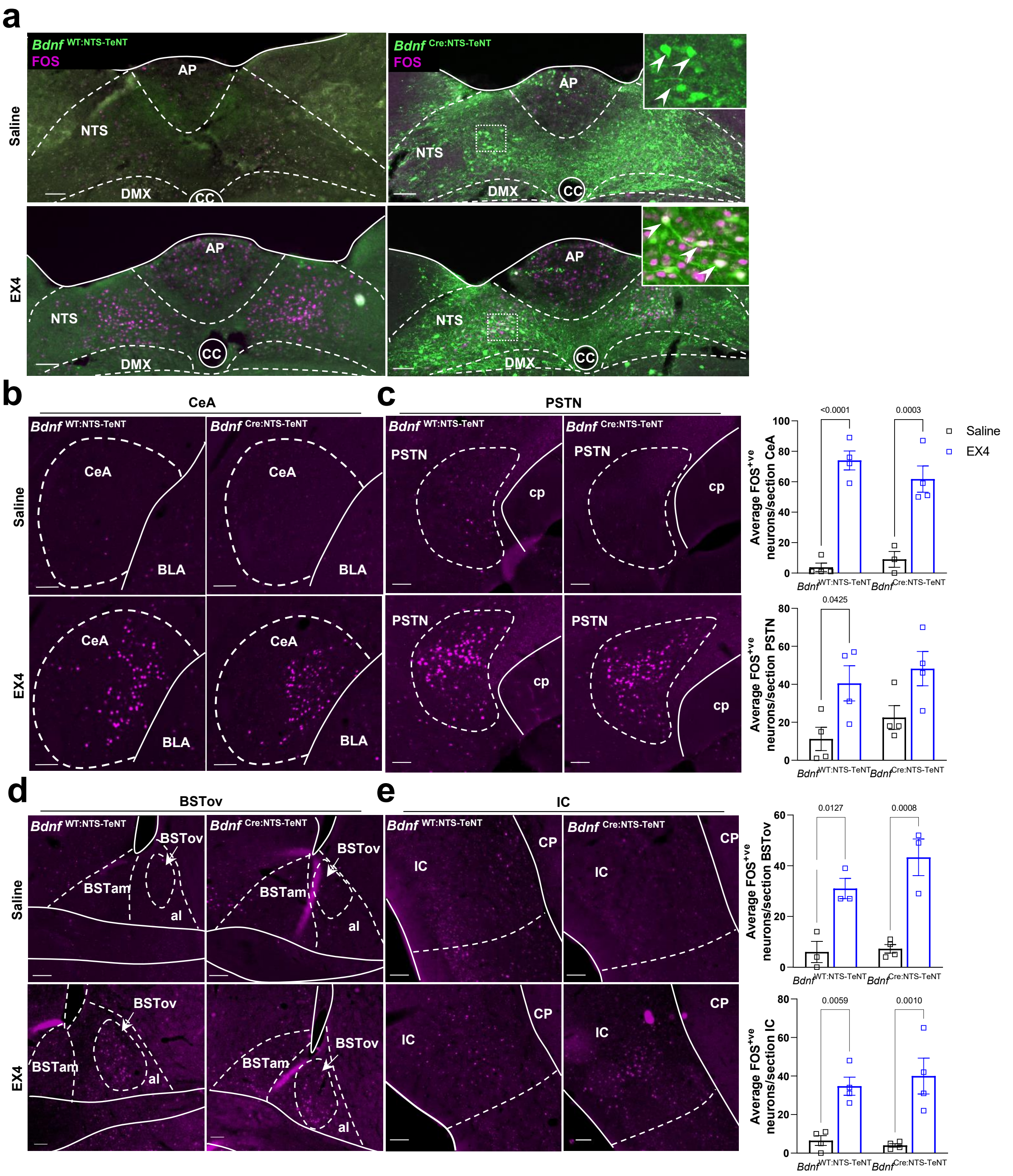

Supplementary Figure 7. Administration of EX4 induces FOS in multiple brain regions. (**a**) Representative immunofluorescence images of FOS (magenta) and AAV-TeNT expression in BDNF^{NTS} neurons (green) of *Bdnf*^{cre} and *Bdnf*^{WT} mice following injection of saline (black squares) or EX4 (30 μg kg^{-1}; blue squares; n = 8 per group). White arrows in higher magnification insets indicate co-labelled cells. (**b-e**) EX4-induced FOS (magenta) in the (**b**) CeA, (**c**) PSTN, (**d**) BSTov and (**e**) IC of *Bdnf*$^{Cre:NTS\text{-}TeNT}$ and *Bdnf*$^{WT:NTS\text{-}TeNT}$ control mice. The average number of FOS+ve neurons/section is presented on the right (n = 3-4 per group; CeA *Bdnf*$^{Cre:NTS\text{-}TeNT}$ ****p< 0.0001 and *Bdnf*$^{WT:NTS\text{-}TeNT}$ ***p=0.0003, PSTN *Bdnf*$^{Cre:NTS\text{-}TeNT}$ *p=0.0425, BSTov *Bdnf*$^{Cre:NTS\text{-}TeNT}$ *p=0.0127 and *Bdnf*$^{WT:NTS\text{-}TeNT}$ ***p=0.0008, IC *Bdnf*$^{Cre:NTS\text{-}TeNT}$ **p=0.0059 and *Bdnf*$^{WT:NTS\text{-}TeNT}$ **p=0.0010; comparisons made using two-way ANOVA

with Sidak's multiple comparisons test). 3V (third ventricle), IC (infralimbic cortex), AP (area postrema), BSTal, am, ov (bed nucleus of the stria terminalis, anterolateral, medial lateral and oval nuclei), CC (central canal), CeA (central amygdala), cp (cerebral peduncle), CP (caudate putamen), DMX (dorsal motor nucleus of the tenth cranial, vagus nerve), NTS (nucleus of the tractus solitarius), PSTN (parasubthalamic nucleus). Scale bars indicate 100 μm. Source data are provided as a Source Data file.
