# Supplementary material for: Brainstem BDNF neurons are downstream of GFRAL/GLP1R signalling
Source: Nat Commun. 2024 Dec 30;15:10749. doi: 10.1038/s41467-024-54367-y (PMC11685588; doi:10.1038/s41467-024-54367-y)
Supplement: Supplementary file 2 — Reporting Summary [file 41467_2024_54367_MOESM2_ESM.pdf]

nature portfolio

Corresponding author(s): Simon M. Luckman

Last updated by author(s): Oct 3, 2024

Reporting Summary

Nature Portfolio wishes to improve the reproducibility of the work that we publish. This form provides structure for consistency and transparency in reporting. For further information on Nature Portfolio policies, see our Editorial Policies and the Editorial Policy Checklist.

Statistics

For all statistical analyses, confirm that the following items are present in the figure legend, table legend, main text, or Methods section.

n/a	Confirmed	
☐	☒	The exact sample size (*n*) for each experimental group/condition, given as a discrete number and unit of measurement
☐	☒	A statement on whether measurements were taken from distinct samples or whether the same sample was measured repeatedly
☐	☒	The statistical test(s) used AND whether they are one- or two-sided<br>*Only common tests should be described solely by name; describe more complex techniques in the Methods section.*
☐	☒	A description of all covariates tested
☐	☒	A description of any assumptions or corrections, such as tests of normality and adjustment for multiple comparisons
☐	☒	A full description of the statistical parameters including central tendency (e.g. means) or other basic estimates (e.g. regression coefficient) AND variation (e.g. standard deviation) or associated estimates of uncertainty (e.g. confidence intervals)
☐	☒	For null hypothesis testing, the test statistic (e.g. *F*, *t*, *r*) with confidence intervals, effect sizes, degrees of freedom and *P* value noted<br>*Give P values as exact values whenever suitable.*
☒	☐	For Bayesian analysis, information on the choice of priors and Markov chain Monte Carlo settings
☒	☐	For hierarchical and complex designs, identification of the appropriate level for tests and full reporting of outcomes
☒	☐	Estimates of effect sizes (e.g. Cohen's *d*, Pearson's *r*), indicating how they were calculated

Our web collection on statistics for biologists contains articles on many of the points above.

Software and code

Policy information about availability of computer code

Data collection	Comprehensive Laboratory Animal Monitoring System Oxymax for Windows software (CLAMS; Columbus Instruments, Columbus, OH, USA) and Phenomaster LabMaster (TSE Systems, Bad Homburg, Germany) were used for metabolic phenotyping.<br>FLIR Research IR software (Teledyne FLIR LLC, Oregano, USA) was used to conduct thermal imaging.<br>Immunofluorescent images were acquired using a 3D-Histech Panoramic-250 microscope slide scanner. Snapshots of the slide scans were taken using the SlideViewer software (3D-Histech). Further images were collected on a Zeiss Axioimager.M2 upright microscope and captured using a Coolsnap HQ2 camera (Photometrics) through Micromanager software v1.4.23.<br>RNAscope in situ images were acquired using an Olympus VS-120 slide scanner.<br>Calcium imaging was visualised using a Axioskop 2 fluorescence microscope (Zeiss) and Prime BSI Express sCMOS camera (Teledyne Photometrics).
Data analysis	Data analysis were performed using Graphpad Prism (v9).<br>Data analysis was assisted with Microsoft Excel (2016).<br>Image processing and analysis was performed using using CaseViewer (2.4.0) and Fiji ImageJ (v1.48)

For manuscripts utilizing custom algorithms or software that are central to the research but not yet described in published literature, software must be made available to editors and reviewers. We strongly encourage code deposition in a community repository (e.g. GitHub). See the Nature Portfolio guidelines for submitting code & software for further information.

Data

Policy information about availability of data

All manuscripts must include a data availability statement. This statement should provide the following information, where applicable:

- Accession codes, unique identifiers, or web links for publicly available datasets
- A description of any restrictions on data availability
- For clinical datasets or third party data, please ensure that the statement adheres to our policy

All data generated in this study are available in the article, supplementary information and the Source Data file. Source data are provided with this paper.

Research involving human participants, their data, or biological material

Policy information about studies with human participants or human data. See also policy information about sex, gender (identity/presentation), and sexual orientation and race, ethnicity and racism.

Reporting on sex and gender	N/A
Reporting on race, ethnicity, or other socially relevant groupings	N/A
Population characteristics	N/A
Recruitment	N/A
Ethics oversight	N/A

Note that full information on the approval of the study protocol must also be provided in the manuscript.

Field-specific reporting

Please select the one below that is the best fit for your research. If you are not sure, read the appropriate sections before making your selection.

☒ Life sciences ☐ Behavioural & social sciences ☐ Ecological, evolutionary & environmental sciences

For a reference copy of the document with all sections, see nature.com/documents/nr-reporting-summary-flat.pdf

Life sciences study design

All studies must disclose on these points even when the disclosure is negative.

Sample size	Sample size was determined based on similar studies in this field and using power analysis (providing at least 80% power to detect effect sizes, with a significance level of 0.05). Sample sizes are stated in the figure legend for each experiment.
Data exclusions	Data from animals with poor viral transfection/missed injections or from misgenotyped animals was not included. Data was excluded from experiments when food was later discovered in the cage bottoms or following an outlier test.
Replication	For calcium imaging at least 2 animals were used as biological replicates. Experiments were performed on individual brain slices from different mice. Details are reported in the manuscript. Each in vivo experiment was performed in several animals (numbers indicated in the manuscript) and data from each individual animal is shown in the figures. Experiments were conducted with calculated sample sizes based on power analyses to determine statistical differences. Studies were often performed in multiple cohorts due to constraints of equipment and cohorts were combined to form the total number of animals in an experiment. For IHC multiple slices for each individual animal were quantified and averaged for each data point (per section).
Randomization	For all experiments animals were assigned to surgical and experimental groups randomly. Where possible treatment injections were randomised on study days allowing for similar body weights within groups, and repeated later in a crossover design. No randomisation was conducted during calcium imaging experiments to facilitate proper data completion.
Blinding	For in vivo studies the experimenter who conducted the analyses was blind to grouping of the animals. Blinding was not possible for calcium imaging as it was not practically possible to blind the experimenter to pharmacological treatments.

Reporting for specific materials, systems and methods

We require information from authors about some types of materials, experimental systems and methods used in many studies. Here, indicate whether each material, system or method listed is relevant to your study. If you are not sure if a list item applies to your research, read the appropriate section before selecting a response.

Materials & experimental systems

n/a	Involved in the study
☐	☒ Antibodies
☒	☐ Eukaryotic cell lines
☒	☐ Palaeontology and archaeology
☐	☒ Animals and other organisms
☒	☐ Clinical data
☒	☐ Dual use research of concern
☒	☐ Plants

Methods

n/a	Involved in the study
☒	☐ ChIP-seq
☒	☐ Flow cytometry
☒	☐ MRI-based neuroimaging

Antibodies

Antibodies used

We report the use of the following antibodies in this study:

Primary antibodies:
Sheep polyclonal anti-GFRAL (dilution 1:200, Cat. # PA5-47769, Thermo Fisher Scientific, MA, USA)
Rabbit polyclonal anti-cFos (dilution 1:2500 or 1:4000, Cat. #190289, Abcam, Cambridge, UK)
Chicken polyclonal anti-GFP (dilution 1:2000. Cat. #13970, Abcam, Cambridge, UK)
Goat anti-mCherry (dilution 1:5000, #AB0040-200, Sicgen, Cantanhede, Portugal)

Secondary antibodies:
Donkey polyclonal anti-sheep Alexa Fluor 594 (dilution 1:1000, Cat.# A11016 Molecular Probes, OR, USA)
Donkey polyclonal anti-chicken Alexa Fluor 488 (dilution 1:1000, Cat.# 703-545-155 Jackson ImmunoResearch, PA, USA)
Donkey polyclonal anti-rabbit Alexa Fluor 488 (dilution 1:1000, Cat.# 711-545-152, Jackson ImmunoResearch)
Donkey polyclonal anti-rabbit Alexa Fluor 594 (dilution 1:1000, Cat.# 711-585-152, Jackson ImmunoResearch)
Biotinylated Donkey polyclonal anti-rabbit (dilution 1:1000, Cat.# 711-065-152, Jackson ImmunoResearch)
Streptavidin Alexa Fluor 594 (dilution 1:1000, Cat.# 016-580-084, Jackson ImmunoResearch)

RNAscope in situ:
Mouse c-fos probe (Cat.# 316928-C3, Advanced Cell Diagnostics, CA, USA)
Mouse Gfral probe (Cat.# 417028-C2, Advanced Cell Diagnostics, CA,USA)
Mouse Bdnf probe (Cat.# 424828-C1, Advanced Cell Diagnostics, CA, USA)

Validation

All antibodies used in this study are commercially available and were validated by manufacturer and/or studies cited by the company websites:

Primary antibodies:
Sheep polyclonal anti-GFRAL (dilution 1:200, Cat. # PA5-47769, Thermo Fisher Scientific, MA, USA)
https://www.thermofisher.com/antibody/product/GFRAL-Antibody-Polyclonal/PA5-47769
Rabbit polyclonal anti-cFos (dilution 1:2500 or 1:4000, Cat. #190289, Abcam, Cambridge, UK)
https://www.abcam.com/en-gb/products/primary-antibodies/c-fos-antibody-bsa-free-ab190289
Chicken polyclonal anti-GFP (dilution 1:2000. Cat. #13970, Abcam, Cambridge, UK)
https://www.abcam.com/en-gb/products/primary-antibodies/gfp-antibody-ab13970
Goat anti-mCherry (dilution 1:5000, #AB0040-200, Sicgen, Cantanhede, Portugal)
https://store.sicgen.pt/catalog/product/AB0040

Secondary antibodies:
Donkey polyclonal anti-sheep Alexa Fluor 594 (dilution 1:1000, Cat.# A11016 Molecular Probes, OR, USA)
https://www.thermofisher.com/antibody/product/Donkey-anti-Sheep-IgG-H-L-Cross-Adsorbed-Secondary-Antibody-Polyclonal/A-11016
Donkey polyclonal anti-chicken Alexa Fluor 488 (dilution 1:1000, Cat.# 703-545-155 Jackson ImmunoResearch, PA, USA)
https://www.jacksonimmuno.com/catalog/products/703-545-155
Donkey polyclonal anti-rabbit Alexa Fluor 488 (dilution 1:1000, Cat.# 711-545-152, Jackson ImmunoResearch)
https://www.jacksonimmuno.com/catalog/products/711-545-152
Donkey polyclonal anti-rabbit Alexa Fluor 594 (dilution 1:1000, Cat.# 711-585-152, Jackson ImmunoResearch)
https://www.jacksonimmuno.com/catalog/products/711-585-152
Biotinylated Donkey polyclonal anti-rabbit (dilution 1:1000, Cat.# 711-065-152, Jackson ImmunoResearch)
https://www.jacksonimmuno.com/catalog/products/711-065-152
Streptavidin Alexa Fluor 594 (dilution 1:1000, Cat.# 016-580-084, Jackson ImmunoResearch)
https://www.jacksonimmuno.com/catalog/products/016-580-084

RNAscope in situ:
Mouse c-fos probe (Cat.# 316928-C3, Advanced Cell Diagnostics, CA, USA)
https://acdbio.com/search/site/%252A316928-C3%252A/cms/probes
Mouse Gfral probe (Cat.# 417028-C2, Advanced Cell Diagnostics, CA,USA)

https://acdbio.com/search/site/%252A417028-C2%252A/cms/probes
Mouse Bdnf probe (Cat.# 424828-C1, Advanced Cell Diagnostics, CA, USA)
https://acdbio.com/search/site/%252A424828%252A/cms/probes/channel/1

Animals and other research organisms

Policy information about studies involving animals; ARRIVE guidelines recommended for reporting animal research, and Sex and Gender in Research

Laboratory animals	All study mice are bred on a C57BL/6J background and strain information is included in the manuscript, as well as below. The age and sex of mice used in each study are clearly described in the methods or figure legends, as well as below. The n numbers for each experiment are clearly reported in the figure legends.<br><br>R26R-EYFP, ChR2-eYFP, BdnfCre and Ai95D (GCaMP6) mice were purchased from Jackson Laboratories (B6.129X1-Gt(ROSA)26Sortm1(EYFP)Cos/J; stock # 006148, B6;129S-Gt(ROSA)26Sortm32(CAG-COP4*H134R/EYFP)Hze/J, stock # 012569, B6.FVB-Bdnfem1(cre)Zak/J stock # 03018 and B6;129S-Gt(ROSA)26Sortm95.1(95.1(CAG-GCaMP6f)Hze/J stock # 024105 respectively; Jackson Laboratories, Maine, USA) and were subsequently bred and crossed with other strains in-house.<br><br>GfralCre and PrlhCre mice were generated by GenOway (GenOway, Lyon, France) and subsequently bred and crossed with other strains in-house.<br><br>For validation of GfralCre:ChR2-eYFP with Gfral antibody and neuronal activation of GDF15 male and female GfralCre:ChR2-eYFP (12 week old) mice were bred in-house.<br>For optogenetic feeding experiments GfralCre:ChR2-eYFP male mice were bred in-house and surgery performed at 8-16 weeks old.<br>For GfralCre:hM3Dq-mCherry chemogenetic studies male and female mice were bred in-house and used at 10-16 weeks old.<br>For RNAscope data male C57BL/6J mice from Janvier (Laval, France) were obtained at 12 weeks old.<br>For calcium imaging male and female GfralCre:Ai95D and BdnfCre:Ai95D mice were bred in-house (10-26 weeks old).<br>For BdnfCre chemogenetic studies male mice were bred in house and surgery performed at 18 weeks old.<br>For BdnfCre neuronal silencing studies male and female mice were bred in house and surgery performed at 10 weeks old.<br><br>For CeA Fluorogold injections male and female CalcaCre::GFP mice were used (kindly donated by Dr Richard Palmiter (B6.Cg-Calcatm1.1(cre/EGFP)Rpa/J, Hughes Medical Institute, University of Washington, USA) and subsequently bred in-house).<br>For PBN and NTS Fluorogold injections 12-14 week old male C57BL/6J mice were obtained from Charles River (Manston, Kent, UK).<br>For neuronal activation of Prlh neurons, PrlhCre:EYFP male mice were bred in house and used at 9-13 weeks old.
Wild animals	No wild animals were used in this study.
Reporting on sex	The sex of all animals is reported in the manuscript.
Field-collected samples	No field-collected samples were used in this study.
Ethics oversight	All animal studies were performed in accordance with the UK Animals and Scientific Procedure Act, 1986, and approved by the local animal welfare ethical review body (AWERB), and in compliance with Eli Lilly and Company's Institutional Animal Care and Use Committee.

Note that full information on the approval of the study protocol must also be provided in the manuscript.

Plants

Seed stocks	N/A
Novel plant genotypes	N/A
Authentication	N/A
